# Minimum Meal Frequency Practice and Its Associated Factors among Children Aged 6–23 Months in Amibara District, North East Ethiopia

**DOI:** 10.1155/2019/8240864

**Published:** 2019-12-18

**Authors:** Mohammed Wagris, Anwar Seid, Molla Kahssay, Osman Ahmed

**Affiliations:** ^1^Department of Public Health, Samara University, Semera, Afar, Ethiopia; ^2^Department of Nursing, Samara University, Semera, Afar, Ethiopia

## Abstract

**Background:**

Minimum meal frequency, a proxy indicator for a child's energy requirements, examines the number of times children received foods other than breast milk. Without adequate diversity and meal frequency, infants and young children are vulnerable to malnutrition, especially stunting and micronutrient deficiencies, and increased morbidity and mortality. In Ethiopia, only 45% of children had fed with an age-appropriate minimum meal frequency.

**Objective:**

The study was aimed to assess the minimal meal frequency practice, and its associated factors among children aged 6–23 months in Amibara district, North East Ethiopia.

**Methods:**

A community-based cross-sectional study was conducted from May 07–May 23, 2018. Systematic random sampling technique was applied to select 367 children aged 6–23 months. The univariable and multivariable binary logistic regression analyses model was used to identify potential predictors of meeting minimum meal frequency. All variables with *P* values <0.25 in the univariable analysis were taken to multivariable analysis, and variables at *P* values <0.05 were considered as statistically significant.

**Results:**

The study revealed a prevalence of minimum meal frequency 69.2% (95% CI: 0.64–0.74). Timely initiation of breastfeeding (AOR = 2.2, 95% CI (1.17, 4.18)), current breastfeeding status (AOR = 7.5, 95% CI (3.95, 14.4)), meeting minimum dietary diversity (AOR = 3.7, 95% CI (1.85, 7.44)), and household hunger scale (AOR = 5.3, 95% CI (1.5, 12.5)) were some of the significant predictors to achieve minimum meal frequency.

**Conclusion:**

The prevalence of minimum meal frequency practice is low in the study area. Current breastfeeding status, timely initiation of breastfeeding, no/little household hunger scale, and meeting minimum dietary diversity were found as significant predictors for minimum meal frequency practice. Mothers having children aged 6–23 months should be aware and practice appropriate infant and young child feeding practices including timely initiation of breastfeeding, breastfeeding till the child celebrate his/her second birthday, recommended meal frequency, and dietary diversity practice. In addition, households should be assessed and strengthened for food security.

## 1. Background

Adequate nutrition during birth to two years of age is a critical window period for the promotion of optimal growth, health, and behavioral development [1]. Around the age of 6 months, an infant's need for energy and nutrients increase. Hence, starting complementary foods are necessary to meet the infant's energy and nutrient requirements. Complementary foods should be introduced at 6 months of age and must be given appropriately, unless, infant's growth may falter [2].

Inappropriate feeding practices are the most risk of malnutrition, illness, and mortality in both infants and young children less than 24 months of age, and more than two-thirds of children deaths related to malnutrition are associated with inappropriate feeding practices during the first 24 months of life [3].

Over 50 million children under age five are wasted, and in low-income countries, one in every three children suffers from stunted growth; in reality, many children never reach this age, and the effects of poor nutrition and stunting continue throughout life, contributing to poor school performance, reduced productivity, and impaired intellectual and social development [4]. The level of malnutrition is high in Ethiopia [5].

Minimum meal frequency, a proxy for a child's energy requirements, examines the number of times children received foods other than breast milk. The minimum number is specific to the age and breastfeeding status of the child. Breastfed children are considered to be consuming minimum meal frequency if they receive solid, semisolid, or soft foods at least twice a day for infants of age 6–8 months and at least three times a day for children of age 9–23 months. Nonbreastfed children aged 6–23 months are considered to be fed with a minimum meal frequency if they receive solid, semisolid, or soft foods at least four times a day [6].

Globally, only a few children are receiving nutritionally sufficient and diversified foods; in many countries, less than one fourth of infants aged 6–23 months meet the criteria for dietary diversity and feeding frequency [7]. The prevalence of minimum meal frequency practice among children aged 6–23 months in Ethiopia is very low, i.e., 45% [6]. There are limited studies regarding minimum meal frequency practice in pastoral community, and so far no similar study was done in the study area; hence, this study aimed to assess the prevalence of minimum meal frequency practice and its associated factors in Amibara district, Afar region, North East Ethiopia.

## 2. Methods

### 2.1. Study Area

This study was conducted in Amibara woreda. Amibara is one of the 32 woredas in Afar regional state. The woreda has bordered on the north by Gewani woreda, on the northwest by Hari Rasu (Samuroobi Galalu woreda) Administrative zone, on the south by awash fentiale woreda, on the east by Ethiopian Somali region, on the southeast by Oromia region, and on the west by Dulecha woreda.

The Woreda is found 279 kms away from Addis Ababa capital city of Ethiopia and 360 kms away from Samara administrative town of Afar regional state. Currently, Amibara woreda has 19 kebelle (the smallest administrative unit in Ethiopia next to district) from this 15 are rural, and the rest 4 kebelles are urban kebelles.

According to 2009, in the annual report of Amibara district, it had a total population of 81,811 of whom 44,178 were men and 37,633 were women, and of them, 8262 and 2, 455 were estimated to be children aged 6–59 months and children aged 6–23 months, respectively.

The district population has pastoral and agropastoral community. The livelihood of the people in the community is irrigation and rain-fed crop production combined with livestock rearing. Cotton- and irrigation-based crop production was mainly practiced in the district. There are one hospital, four health centers, and twenty functional health posts in the district.

### 2.2. Study Design and Period

A community based cross-sectional study was conducted from May 7 to May 23, 2018.

### 2.3. Eligibility Criteria

All mothers having children aged 6–23 months were included in the study. Mothers having children aged 6–23 months, who were not able to respond the interviews due to illness, were excluded from the study.

### 2.4. Sample Size Determination

Sample size was calculated using a formula for a single population proportion considering 95% confidence level, 80% power, 5% margin of error, 68.4% maximum variability (prevalence of minimum meal frequency from a research conducted in South East Ethiopia [8]), and 10% nonresponse. The sample size was 333, and after adding 10% nonresponse rate, the final sample size was 367.

(1) $$\begin{array}{c} n=\frac{{\left(Z_{\alpha /2}\right)}^{2}*P*\left[1-P\right]}{d^{2}},\\ n=\frac{{\left[1.96\right]}^{2}*0.684*\left[0.316\right]}{{\left[0.05\right]}^{2}}=333. \end{array}$$

Upon adding 10% nonresponse rate ((333*∗*10%) + 333), the final sample size was 367.

### 2.5. Sampling Procedure

Simple random sampling technique was used to select six rural and two urban kebelles. Population proportion to size was used to estimate the number of samples from each kebelle. Finally, systematic random sampling technique was applied to select the study participants after a total of 1034 infants, and young children aged 6–23 months were obtained.

### 2.6. Data Collection Tools

The data collection tools were adopted from EDHS 2016 questionnaire with some modification and questionnaire assessing meal frequency and was adopted from WHO standardized questionnaire for infant and young child feeding (IYCF) practices.

### 2.7. Data Collection Procedure

Primarily questionnaire was formulated in English language. The English version questionnaire was translated to local language which is Afar'af language and then translated back to English to check for consistency. Eight data collectors and two supervisors were recruited to handle the overall data collection process.

### 2.8. Operational Definitions of Terms

#### 2.8.1. Minimum Meal Frequency

For the proportion of breastfed and nonbreastfed children who are 6–23 months of age and who receive solid, semisolid, or soft foods (but also including milk feeds for nonbreastfed children), the minimum is defined as: 2 times for breastfed infants aged 6–8 months, 3 times for breastfed children aged 9–23 months, and 4 times for nonbreastfed children aged 6–23 months [9].

#### 2.8.2. Minimum Dietary Diversity Score

It is the proportion of children who are 6–23 months of age and who receive foods from 4 or more food groups [9].

### 2.9. Data Quality Control

A local language (Afar'af) was used to collect the data. Data collectors were local language speakers. Pretest was done in 10% of the total samples. Pretest was done in nonselected kebelles of the woreda. Two days training was given for the data collectors and supervisors. Routine daily checkup was done by supervisors and principal investigator in data collection period.

### 2.10. Data Processing and Analysis

After the end of data collection, data were checked for completeness, entered in to Epi-data version 3.1 for cleaning and transported to SPSS version 20 for analysis. Descriptive analysis was used to see frequency and percentages of the characteristics. Binary logistic regression was used to assess significant predictors of the outcome variable. Variables having *P* value <0.25 in univariable binary logistic regression were taken to multivariable binary logistic regression to control the confounding effect. Finally, odds Ratio (OR) and 95% confidence intervals (CI) were used to express the final mode, and statistical significance was declared at *P* value ≤0.05.

### 2.11. Ethical Consideration

Ethical clearance was obtained from Samara University Ethical Review Committee. Letter of support was obtained from the Afar Regional Health Bureau, Amibara woreda health office. Informed oral consent was also obtained from study participants.

## 3. Results

### 3.1. Sociodemographic Characteristics of Study Subjects

A total of 364 mother-child pairs with a response rate of 99.18% were included in the analysis. The mean age of mothers was 26.4(±5.40) years. More than half of study subjects were of age 20–34 years. Regarding educational status, about 44% of mothers were unable to read and write. 47.8 and 44.5 percent of the households are 3-4 and above 5 family members. Of the total study subjects, 297 (81.6%) were married, 255 (70.1%) were Muslim by religion. More than half of the respondents were Afar ethnic group, and two hundred forty eight (68.1%) were rural inhabitants (Table 1).

### 3.2. Knowledge of Mothers on Infant and Young Child Feeding

Regarding the knowledge of mothers about child feeding, ten knowledge questions were provided. Participants who scored seven and above, 299 (82.1%) were considered as knowledgeable. Among 364 study participants interviewed, 315 (86.5%) of the mothers had heard of feeding diversified food to their children. Majority of the mothers (87.4%) knew that complementary foods should be introduced at six months of child age. With regard to dietary diversity, 321 (88.2%) mothers confirmed that a child should consume at least four types of food groups. 293 (80.5%) the mothers stated that even if a child did not feel hungry, it does not mean that his/her nutritional requirement is fulfilled. 283 (77.7%) of the mothers/caregivers recognized that giving only animal source food is not adequate for child growth and development (Table 2).

### 3.3. Dietary Practice of Study Subjects

Two hundred and sixty two (72%) children were breastfeeding during the interview. All study subjects had ever breastfed their children, and 239 (65.7%) had initiated breastfeeding within the first one hour of birth, but the rest one-third 34.3% mothers had started breastfeeding after one hour of birth. Around 138 (37.9%) had practiced prelacteal feeding and out of this, 35.5%, 32.6%, and 29.7% were given water, butter, and milk, respectively. Nearly one-third (29.9%) of children experienced illness 2 weeks prior to the survey (Table 3).

### 3.4. Minimum Meal Frequency Practice

The overall minimum meal frequency was practiced in 69.2% (95% CI: 0.64, 0.74%) of the participants. To compute minimum meal frequency, infants were categorized into currently breastfeeding (72%) and currently nonbreastfeeding (28%). Age categorization of infants was also used to compute minimum meal frequency. Based on that, 53 (91.4%) and 164 (80.4%) of currently breastfeeding infants aged 6–8 months 9–23 months met the minimum meal frequency, respectively. Regarding nonbreastfeeding infants 24 hrs prior to this survey, only 35 (34.3%) met minimum meal frequency (Table 3).

### 3.5. Maternal Health Care Utilization Characteristics of Study Subjects

Regarding health service utilization, 40% of the study subjects had antenatal care visit for more than three times, and 64.6.% of the study subjects had postnatal care visit. About 223 (76.4%) and 206 (87.7%) of mothers received nutrition counseling on infant and young child feeding practices by health professionals during their antenatal and postnatal visit, respectively. Around seventy percent of mothers gave birth at health institutions (Table 4).

### 3.6. Factors Associated with Minimal Meal Frequency Practice

In univariable binary logistic regression, residence, place of delivery, antenatal care visit, postnatal care visit, maternal education, timely initiation of breastfeeding, current breastfeeding status, source of food, household hunger scale, meat minimum dietary diversity, family size, and media exposure were some of the determinant factors (with *P* value <0.25) for meeting minimum meal frequency. Those variables having the *P* value of <0.25 in univariable analysis were taken to multivariable binary logistic regression analysis. In multivariable binary logistic regression analysis, those variables with *P* value <0.05 were considered significant predictors to meet minimum meal frequency. Therefore, timely initiation of breastfeeding, current breastfeeding status, meeting minimum dietary diversity, and household hunger scale were some of the predictor variables with *P* value <0.05. Respondents who had experienced timely initiation of breastfeeding were 2 times more likely to meet minimum meal frequency comparing with their counterparts, AOR = 2.2, 95% CI (1.17, 4.18). Currently, breastfeeding children were 7.5 times more likely to achieve minimum meal frequency comparing with their counterparts, AOR = 7.5, 95% CI (3.95, 14.4). Children who had met minimum dietary diversity score were 3.7 times more likely to achieve minimum meal frequency comparing with their counterparts, AOR = 3.7, 95% CI (1.85, 7.44). Children from household with no and little household hunger scale were 5 times more likely to achieve minimum meal frequency comparing with children from households with moderate-to-severe hunger scale, AOR = 5.3, 95% CI (1.5, 12.5) (Table 5).

## 4. Discussion

The prevalence of children aged 6–23 months who received the recommended minimum meal frequency were 69% (95% CI: 0.64–0.74). The minimum meal frequency practice was nearly similar with the study conducted in Bale Zone, South West Ethiopia (68.4%) [8], but less than from Bangladesh (81%), Colombia (72%), Bolivia (74%), and Madagascar (76%), [10]. This difference might be due to the livelihood nature of the pastoral community and household food insecurity.

The minimum meal frequency practice was higher than the finding from Ethiopian demographic and health survey (45%) [6], Dangila town northwest Ethiopia (50.4%) [11], Assella town South East Ethiopia (53.8%) [12], Egypt (58%) [10], Ghana (46%) [13], and northern Ghana (57.3%) [14]. This might be due to a difference in the study period, and sociodemographic characteristics of study population.

Regarding factors associated with achieving minimum meal frequency, respondents who had experienced timely initiation of breastfeeding were 2 times more likely to meet minimum meal frequency comparing with their counterparts, AOR = 2.2, 95% CI (1.17, 4.18). This can be due to that mothers who had appropriate infant and young child feeding practice (timely initiation of breastfeeding) are more likely to continue such positive and appropriate practice including feeding their children with appropriate meal frequency. Besides, mothers who had a positive experience are thought to have a previous exposure of appropriate practices.

Currently, breastfeeding children were 7.5 times more likely to achieve minimum meal frequency comparing with their counterparts, AOR = 7.5, 95% CI (3.95, 14.4). This can be possibly because the meal frequency of breastfeeding children decreases comparing with those nonbreastfeeding children, and nonbreastfeeding children are requested to eat at least four meals per day, but there is at least one meal reduction in breastfeeding children to fulfill the criteria for achieving minimum meal frequency.

Children who had met minimum dietary diversity score were 3.7 times more likely to achieve minimum meal frequency comparing with their counterparts, AOR = 3.7, 95% CI (1.85, 7.44). This is due to that households with the availability of diversified food are more likely to feed their children frequently. Availability of food is one core criterion to achieve minimum meal frequency.

Children from household with no and little household hunger scale were 5 times more likely to achieve minimum meal frequency comparing with children from households with moderate-to-severe hunger scale, AOR = 5.3, 95% CI (1.5, 12.5). This might be due to low purchasing capacity of households for food items. If food is not available in households, it is too tough for the children to get a required number of meals.

## 5. Limitation of the Study

Due to the fact that the study was a cross-sectional study, describing cause and effect relationship of the exposure and outcome variables is difficult.

## 6. Conclusion

The prevalence of minimum meal frequency practice is low in the study area. Current breastfeeding status, timely initiation of breastfeeding, no/little household hunger scale, and meeting minimum dietary diversity were found as significant predictors for minimum meal frequency practice. Mothers having children aged 6–23 months should be aware and practice appropriate infant and young child feeding practices including timely initiation of breastfeeding, breastfeeding till the child celebrate his/her second birthday, recommended meal frequency, and recommended dietary diversity practice. In addition, households should be assessed and strengthened for food security.

## Figures and Tables

**Table 1:** Sociodemographic and economic characteristics of study subjects in Amibara district, North East Ethiopia, 2018 (*n* = 364).

Variable	Category	Frequency	%
Age of the child (in months)	6–8	60	16.5
	9–23	304	83.5

Sex of the child	Male	194	53.3
	Female	170	46.7

Birth order of the child	1^st^	117	32.1
	2^nd^-3^rd^	162	44.5
	≥4^th^	85	23.4

Maternal age (in years)	<20 years	52	14.3
	20–34 years	283	77.7
	>34	29	8.0

Religion	Muslim	255	70.1
	Orthodox	77	21.2
	Protestant	30	8.2
	Other	2	0.5

Maternal marital status	Married	297	81.6
	Single	10	2.7
	Divorced	42	11.5
	Widowed	15	4.1

Household family size	2	28	7.7
	3–4	174	47.8
	≥5	162	44.5

Average HH monthly income	<1000	106	29.1
	≥1000	258	70.9

**Table 2:** Knowledge of mothers on infant and young child feeding among children aged 6–23 months in Amibara district, North East Ethiopia, 2018 (*n* = 364).

Knowledge variables	Frequency	%
Heard about importance of feeding diversified foods to a 6–23-month child	315	86.5
Complementary feeding should start at 6 months of child age	318	87.4
A 6–23-month child should eat four or more food groups	313	86
Giving meat is advisable for a 6–23- month child	283	77.7
One cause of childhood malnutrition is not having diversified foods	321	88.2
Did not feel hungry does not mean that the nutritional need of a child is fulfilled	293	80.5
One cause of childhood malnutrition is not starting complementary feeding at 6 months of child age	317	87.1
Feeding only animal products is not enough/adequate for a 6–23-month child	313	86
A 6–23- month child should feed organ meat, such as liver and kidney	237	65.1
A 6–23-month child should feed an egg	301	82.7

*Overall knowledge score*		
Good knowledge	299	82.1
Poor knowledge	65	17.9

**Table 3:** Breastfeeding and meal frequency practice among children aged 6–23 months in Amibara district, North East Ethiopia, 2018 (*n* = 364).

Characteristics	Category	Frequency	Percentage
Prelacteal feeding	Yes	138	37.9
	No	226	62.1

Currently breastfeeding	Yes	262	72
	No	102	28

Child history of illness in the past 2 weeks	Yes	109	29.9
	No	255	70.1

Timely initiation of breastfeeding	Yes	239	65.7
	No	125	34.3

Minimum meal frequency 6–8 months and currently breastfeeding meal >2 times	Yes	53	91.4
	No	5	8.6

Minimum meal frequency 9–23 months and currently breastfeeding meal >3 times	Yes	164	80.4
	No	40	19.6

Minimum meal frequency 6–23 months and currently not breastfeeding meal >4 times	Yes	35	34.3
	No	67	65.7

Overall minimum meal frequency (number of minimum meals for their age)	Yes	252	69
	No	112	31

**Table 4:** Maternal health care utilization characteristics of mothers having children aged 6–23 months in Amibara district, North East Ethiopia, 2018 (n = 364).

Variable	Category	Frequency	%
Parity of mother	1	113	31
	2–4	194	53.3
	≥5	57	15.7

History of antenatal care visit	Yes	292	80.2
	No	72	19.8

Frequency of antenatal care visit (*n* = 292)	1^st^ visit	3	0.8
	2-3 visit	142	39
	≥4	147	40.4

Counseling on infant and young child feeding during ANC visit (*n* = 292)	Yes	223	76.4
	No	69	23.6

Postnatal care service visit	Yes	235	64.6
	No	129	35.4

Counseling on infant and young child feeding during PNC visit (*n* = 235)	Yes	206	87.7
	No	29	12.3

Place of delivery	Home	108	29.7
	Health institution	256	70.3

**Table 5:** Binary logistic regression analysis for determinants of meeting minimum meal frequency among children aged 6–23 months in Amibara district, North East Ethiopia, Ethiopia, 2018 (*n* = 364).

Variables		Meet minimum meal frequency		COR (95% CI)	AOR (95% CI)
		Yes	No		
Residence	Urban	86	30	1.42 (0.87, 2.32)	0.69 (0.33, 1.41)
	Rural	166	82	1	1

Place of delivery	Home	63	45	1	1
	Health institution	189	67	2 (1.26, 3.23)^*∗*^	1.35 (0.55, 3.32)

ANC service visit	Yes	211	81	1.97 (1.12, 3.34)^*∗*^	1.8 (0.69, 4.7)
	No	41	31	1	1

PNC service visit	Yes	173	62	1.77 (1.12, 2.79)^*∗*^	1.5 (0.65, 3.7)
	No	79	50	1	1

HH hunger scale	Little to no hunger	247	93	10.1 (3.67, 17.4)^*∗*^	5.3 (1.5, 12.5)^*∗*^
	Severe to moderate hunger	5	19	1	1

Early initiation of breastfeeding	Yes	195	44	5.29 (3.27, 8.5)^*∗*^	2.2 (1.17, 4.18)^*∗*^
	No	57	68	1	1

Media exposure	Yes	168	52	2.3 (1.47, 3.64)^*∗*^	1.32 (0.65, 2.65)
	No	84	60	1	1

Currently breastfeeding	Yes	217	45	9.23 (5.49, 15.5)	7.5 (3.95, 14.4)^*∗*^
	No	35	67	1	1

Source of food	Own production	15	7	4 (1.36, 11.9)^*∗*^	4.61 (0.95, 13.4)
	Purchase	221	75	5.53 (2.85, 10.7)^*∗*^	1.94 (0.76, 4.95)
	Food aid	16	30	1	1

MDDS	Yes	141	95	4.4 (2.48, 7.8)^*∗*^	3.71 (1.85, 7.44)^*∗*^
	No	111	17	1	1

Note: ^*∗*^*P* value <0.05; COR: crude odds ratio; AOR: adjusted odds ratio.

## Data Availability

The datasets supporting the conclusions of the study are included in the article. Any additional data will be available on request. The datasets used and/or analyzed during the current study are available from the corresponding author (Molla) upon reasonable request.

## Abbreviations

IYCF: Infant and young child feeding
MDDS: Minimum dietary diversity score
MMF: Minimum meal frequency
WHO: World Health Organization
SPSS: Statistical Package for the Social Science.
